# Digital Interventions for Combating Internet Addiction in Young Children: Qualitative Study of Parent and Therapist Perspectives

**DOI:** 10.2196/55364

**Published:** 2024-04-26

**Authors:** Yansen Theopilus, Abdullah Al Mahmud, Hilary Davis, Johanna Renny Octavia

**Affiliations:** 1 Centre for Design Innovation Swinburne University of Technology Melbourne Australia; 2 Centre for Ergonomics Parahyangan Catholic University Bandung Indonesia; 3 Centre for Social Impact Swinburne University of Technology Melbourne Australia

**Keywords:** addiction therapist, children, digital intervention, internet addiction, digital devices, parents, parental control, mobile phone

## Abstract

**Background:**

Internet addiction is an emerging mental health issue in this digital age. Nowadays, children start using the internet in early childhood, thus making them vulnerable to addictive use. Previous studies have reported that the risk of internet addiction tends to be higher in lower-income regions with lower quality of life, such as Indonesia. Indonesia has high risks and prevalence of internet addiction, including in children. Digital interventions have been developed as an option to combat internet addiction in children. However, little is known about what parents and therapists in Indonesia perceive about these types of interventions.

**Objective:**

This study aims to investigate the experiences, perceptions, and considerations of parents and therapists regarding digital interventions for combating internet addiction in young Indonesian children.

**Methods:**

This study used a qualitative exploratory approach through semistructured interviews. We involved 22 parents of children aged 7 to 11 years and 6 experienced internet addiction therapists for children. The interview data were transcribed and analyzed using thematic analysis.

**Results:**

Participants in this study recognized 3 existing digital interventions to combat internet addiction: Google Family Link, YouTube Kids, and Apple parental control. They perceived that digital interventions could be beneficial in continuously promoting healthy digital behavior in children and supporting parents in supervision. However, the existing interventions were not highly used due to limitations such as the apps’ functionality and usability, parental capability, parent-child relationships, cultural incompatibility, and data privacy.

**Conclusions:**

The findings suggest that digital interventions should focus not only on restricting and monitoring screen time but also on suggesting substitutive activities for children, developing children’s competencies to combat addictive behavior, improving digital literacy in children and parents, and supporting parental decision-making to promote healthy digital behavior in their children. Suggestions for future digital interventions are provided, such as making the existing features more usable and relatable, investigating gamification features to enhance parental motivation and capability in managing their children’s internet use, providing tailored or personalized content to suit users’ characteristics, and considering the provision of training and information about the use of interventions and privacy agreements.

## Introduction

### Background

Digital devices and the internet have useful functions for supporting our daily lives and work. Multiple studies have reported that, nowadays, children start using digital devices in early childhood [1-5]. Digital device ownership has also increased rapidly among young children [6,7]. Providing children with access to the internet can be beneficial for them and their parents, especially for learning and entertainment purposes [8,9]. However, increasing internet use in young children is followed by increasingly urgent risks that they will not be able to self-manage their digital behavior wisely. The addictive use of the internet and digital devices has emerged as one of the most anticipated concerns related to internet use in young children [7,10].

Internet addiction (IA) is defined as a behavioral disorder caused by the excessive and uncontrolled use of the internet and digital devices that can have negative impacts on mental, physical, and social health [11]. Behavioral addiction related to the internet and gaming has been recognized as a diagnosable mental health condition that needs further research in the *International Statistical Classification of Diseases and Related Health Problems, 11th Revision*, and the *Diagnostic and Statistical Manual of Mental Disorders, 5th Edition* [12,13]. IA can have harmful consequences for young children, such as speech delay, physical disorders, personality disorders, aggressive behavior, eating disorders, self-isolation, decreased academic performance, and decreased vision [14-19]. Young children are vulnerable to IA due to their limited self-control, limited digital literacy, incomplete cognitive development, and influence from family and their environment [20-23].

According to the Interactional Theory of Childhood Problematic Media Use (IT-CPU), the development of IA risks in young children (aged <12 years) could be defined from combined psychology, communication, and human-computer interaction perspectives [7]. On the basis of the IT-CPU, the problem in children is jointly influenced by distal factors (eg, the family’s socioeconomic condition, the family’s dysfunction, and the digital environment), proximal factors (eg, children’s behavior and emotion; the family’s behavior, attitude, literacy, and media use; and peers’ technology access), and maintaining factors (eg, parent-child relationships, children’s media use engagement and motivation, and peer influence) [7].

Some approaches to combat IA in young children include education, therapy, digital parenting, strategic physical activity, and digital intervention [24,25]. Although digital interventions are sometimes seen as an effort to “fight fire with fire,” previous studies have reported that they might have the potential to combat IA due to the ability to use technology to promote healthy digital behavior with lower effort [25-27]. Multiple studies have also reported that digital interventions show promising efficacy for combating smartphone addiction in adolescents or adults [28-31].

Some types of digital interventions are common to promote healthy digital behavior, such as parental control and digital well-being software. They offer various functions, such as screen time monitoring and limitations, app management, content restrictions, and location tracking. Parental control software aims to support parents in monitoring and regulating children’s devices remotely (eg, Google Family Link and Apple parental control) [32]. Digital well-being software supports the user in self-monitoring and self-limiting the use of the internet and digital devices [26]. Digital well-being systems are available in stand-alone apps (eg, ActionDash and StayFree) or integrated within operating systems (eg, Android and iOS), devices (eg, Samsung and Oppo), and apps (eg, TikTok, Facebook, and YouTube).

The research and development of digital interventions for young children is still in its infancy [25]. A previous study in South Korea reported that the efficacy of parental control software was not promising in terms of reducing addictive behavior in children [25,33]. In addition, it is not known how digital interventions are currently being used to combat IA in young children. This raises questions about the perceptions and considerations of the people involved in efforts to combat IA in young children and their views on the use of digital interventions. Parents play a vital role in using digital interventions to manage their children’s behavior. IA therapists may also recommend digital interventions to their clients or recommend using them to support IA treatment [34].

Multiple studies have reported that people in lower-income regions (eg, the Eastern Mediterranean, Southeast Asia, and Africa) with a lower quality of life tend to have a higher prevalence of IA [35,36]. In line with those studies, Indonesia is among the countries in Southeast Asia with a high prevalence of IA, including in children [36,37]. Indonesia is a lower-income country with >212 million active internet users, and >30 million of them are children [38,39]. Therefore, this study investigated the use of digital interventions in the Indonesian context as a lower-income country with high risks and prevalence of IA.

Some digital interventions for children are available in Indonesia, such as Google Family Link, Apple parental control, Norton Family, FamiSafe, and Safe Lagoon. However, little is known about the perceptions on digital interventions of children’s stakeholders in Indonesia who are involved in efforts to combat IA. Therefore, this qualitative exploratory study aimed to investigate the experiences, perceptions, and considerations of parents and child IA therapists regarding the use of digital interventions for combating IA in young Indonesian children. We formulated 3 research questions (RQs) to achieve this objective:

1. What are the experiences of parents and therapists in Indonesia with digital interventions to combat IA in young children? (RQ 1)
2. What are the limitations perceived by parents and therapists in Indonesia of digital interventions to combat IA in young children? (RQ 2)
3. What functions are recommended by parents and therapists in Indonesia for digital interventions to combat IA in young children? (RQ 3)

This study contributes to extending the knowledge from parents’ and therapists’ perspectives on the current state, existing perceptions, and future implications of digital interventions to combat IA in young Indonesian children. The findings of this study will be valuable considerations in evaluating the existing interventions and developing better interventions in the future.

### Theoretical Framework

The theoretical framework that underlies the RQs in this study is related to the development and evaluation of digital interventions to combat IA in children. This includes digital behavior change interventions (DBCIs), parental mediation, and the Unified Theory of Acceptance and Use of Technology (UTAUT).

According to DBCIs, digital technologies can be used to support health-related behavior change and promote healthy behavior [40]. Using behavior change theories, models, and frameworks in developing digital health interventions can help the design team address the problem effectively [41]. The use of DBCIs can also be beneficial to combat addictive behavior related to the internet and digital devices [27]. Therefore, this approach may be applied to develop digital interventions for combating IA in young children.

Parental mediation theory emphasizes the parents’ role in communicating about digital media use with children to mitigate negative impacts [42]. This theory suggests active mediation, restrictive mediation, and coviewing as parental strategies to prevent the harmful effects of digital media on children [42]. The original strategy was refined into 4 mediation activities to adapt to the rapid development of the digital media landscape: gatekeeping (regulation), discursive (discussion), investigative (monitoring), and diversionary (alternative activities) [43]. This theory can be used to support parents in combating IA in their children through digital interventions.

The UTAUT model suggests factors that influence the acceptance of the use of digital health interventions [44]. The UTAUT model is a modification of the technology acceptance model that focuses on digital health interventions [45]. According to this model, performance expectancy, effort expectancy, social influence, facilitating conditions, and internet anxiety can jointly influence the acceptance of an intervention [46]. This model underlies the need to investigate the experiences and perceptions of potential users and health practitioners to increase the acceptance of digital interventions for combating problems.

## Methods

### Study Design

This study used a qualitative exploratory approach through semistructured interviews to conduct a detailed exploration of the experiences, perceptions, and considerations of digital interventions to combat IA in children [47]. This approach is commonly used to explore stakeholders’ perspectives on digital health care interventions [48-50]. Previous studies on IA interventions have also emphasized the need to explore the potential, needs, and considerations regarding digital interventions to improve our efforts to combat problematic internet use in children [51-53]. It is essential to investigate this from the perspectives of children’s stakeholders who may have a significant contribution or influence in combating IA in children, such as parents and IA therapists for children. However, little is known about their perceptions and experiences regarding the use of digital interventions to combat IA in children. Therefore, this study contributed to an in-depth investigation of their perspectives through a qualitative exploratory approach to fill the gaps.

This study was systematically reported according to the COREQ (Consolidated Criteria for Reporting Qualitative Research) checklist [54]. This checklist consists of 32 items to report important criteria in qualitative research, such as interviews or focus group discussions (Multimedia Appendix 1 [54]). The researchers of this study comprised a PhD candidate and senior researchers (PhDs) from Australia and Indonesia from various cultures and interdisciplinary backgrounds (eg, human-computer interaction, social science, and product design). We have previous experience in digital intervention research for special populations (eg, children, older adults, and people with mental health conditions).

### Participants

This study involved 2 stakeholders who have important roles in combating IA in young children, including parents as the primary guardians of their children and IA therapists for children. Parents are typically the main actors who provide digital devices and supervision to their children [55,56]. Therefore, they are responsible for ensuring their children’s digital health and well-being. Child addiction therapists in Indonesia are psychologists or psychiatrists who have expertise and experience in working with children with IA risks. They also have a duty to promote the prevention of addictive use. Involving parents and child therapists provided valuable and comprehensive insights into the experiences and considerations of using digital interventions to combat IA in young children.

A total of 28 participants (n=22, 79% parents and n=6, 21% experienced therapists) took part in this study. The inclusion criteria for parents were (1) being the primary guardians of children aged 7 to 11 years, (2) both parents and children being active internet users, and (3) residing with their children. If the parents had more than one child, we asked them to focus on one child who met our inclusion criteria when participating in this study. This was to ensure their consistency in sharing their experience with their children. They were recruited using a convenience sampling strategy through parenting communities in Indonesia. We sent invitations to 20 parenting communities in Indonesia to participate in this study through web-based platforms such as Facebook, Instagram, and WhatsApp. We provided the researcher’s contact details (YT) on the invitation so that parents could express their willingness to participate. Of the 33 parents who were initially interested in participating, 11 (33%) refused to participate for personal reasons after knowing the procedure and goals of this study. At the beginning of data collection, we conducted a preliminary test using the Parent-Child Internet Addiction Test (PCIAT) to obtain previous knowledge about whether their children might have a normal, mild, moderate, or severe risk of IA [57]. The PCIAT is a 20-item validated questionnaire to assess children’s IA risks through their primary guardian’s perspective [58].

Similar to the parents, we recruited therapists through a convenience sampling strategy. The inclusion criteria for the therapists were (1) psychologists or psychiatrists with expertise in IA therapy for children and (2) formally recognized therapists with >4 years of experience. We contacted 10 prominent hospitals and psychology bureaus in Indonesia that offer services related to IA treatment for children. This aimed to obtain information about potential therapists that might meet our inclusion criteria. Initially, we invited 12 therapists, but 6 (50%) refused to participate for personal reasons. The characteristics of the participants in this study are shown in Tables 1 (parents) and 2 (therapists).

**Table 1:** Parents’ sociodemographic characteristics (N=22).

Characteristic			Values
Age (y), mean (SD)			36.1 (5.8)
**Gender, n (%)**			
	Women	20 (91)	
	Men	2 (9)	
**Educational level, n (%)**			
	High school	9 (41)	
	Bachelor’s degree	12 (55)	
	Master’s degree	1 (5)	
**Occupation, n (%)**			
	Private employee	6 (27)	
	Stay-at-home parent	9 (41)	
	Entrepreneur	5 (23)	
	Medical practitioner	1 (5)	
	Teacher	1 (5)	
**Family location, n (%)**			
	West Java	12 (55)	
	Central Java	3 (14)	
	East Java	2 (9)	
	Jakarta	2 (9)	
	South Sumatra	2 (9)	
	West Sumatra	1 (5)	
**Family monthly income, n (%)**			
	<IDR^a^ 5 million (<Aus $500 or <US $314.44)	12 (55)	
	IDR 5-30 million (Aus $500-$3000 or US $314.44-$1886.61)	7 (32)	
	>IDR 30 million (>Aus $3000 or >US $1886.61)	3 (14)	
**Number of children, n (%)**			
	1	8 (36)	
	2	8 (36)	
	3	4 (18)	
	4	2 (9)	
Child’s age (y), mean (SD)			8.6 (1.4)
Child’s gender (women), n (%)			8 (36)
**Child’s order of birth, n (%)**			
	First	14 (64)	
	Second	6 (27)	
	Third	2 (9)	
**Devices used by the child, n (%)**			
	Mobile devices (smartphone or tablet)	22 (100)	
	Television	18 (82)	
	Laptop or PC	6 (27)	
	PlayStation console	2 (9)	
**Age when the child first used the internet (years), n (%)**			
	1	8 (36)	
	2	7 (32)	
	3	5 (23)	
	5	2 (9)	
**Child’s PCIAT^b^ risk category, n (%)**			
	Normal	8 (36)	
	Mild	6 (27)	
	Moderate	6 (27)	
	Severe	2 (9)	

^a^IDR: Indonesian rupiah.

^b^PCIAT: Parent-Child Internet Addiction Test.

**Table 2:** Therapists’ sociodemographic characteristics (N=6).

Characteristic		Values
**Gender, n (%)**		
	Women	6 (100)
	Men	0 (100)
Work experience (y), range		5-14
**Location, n (%)**		
	West Java	2 (33)
	Jakarta	2 (33)
	Central Java	1 (17)
	East Java	1 (17)
**Work title, n (%)**		
	Child or clinical psychologist	3 (50)
	Child psychiatrist	3 (50)
**Workplace, n (%)**		
	Public hospital	2 (33)
	Private hospital	2 (33)
	Psychology bureau	2 (33)

### Data Collection

Data collection for this study was conducted from September 2023 to November 2023. As the participants were located in different cities in Indonesia, we conducted the interviews on the web using Microsoft Teams (Microsoft Corporation). Each interview session was conducted by the first author (YT; male) in Bahasa (the national Indonesian language) and lasted 30 to 60 minutes. There was no previous relationship between the interviewer and the participants in this study. Before the interview session, the interviewer explained the objective and scope of this study to each participant. The main topic discussed in the semistructured interviews was the experiences, perceptions, and recommendations regarding using digital interventions to combat IA in children. If the participants were no longer using the digital interventions, they could also share their past experiences and why they stopped using them.

The probing questions of the interviews are shown in Multimedia Appendix 2.

We recorded the audio of each interview session. The recordings were transcribed in Bahasa using Google Speech-to-Text and then manually refined by a researcher (YT) who is a native speaker of Bahasa. The interview transcripts were returned to each participant for checking and correction. After that, we translated them into English for data analysis and reporting purposes.

### Data Analysis

The data analysis was conducted qualitatively using thematic analysis through five main stages: (1) transcribing, reading, and understanding the data; (2) coding the data; (3) identifying meaningful patterns within the data; (4) defining and grouping the themes; and (5) reporting the findings according to the themes [59]. The NVivo (version 12.0; QSR International) software was used to support coding the data and identifying meaningful patterns within the data. Although the thematic analysis did not rely on quantifiable measures, we presented the frequency of themes, subthemes, or issues discussed as additional information to describe the findings and increase reporting transparency [60,61].

To ensure the quality of the data analysis, we followed the 15 criteria for good thematic analysis proposed by Braun and Clarke [60]. The criteria include the transcription (1 item), coding (5 items), analysis (4 items), overall (1 item), and reporting (4 items) processes [60]. The interview transcripts were checked by the interviewer (YT) and participants to ensure their accuracy. The codes and themes were identified and checked rigorously to ensure the validity and consistency of the analysis. The methods, data, and findings of this study were described and reported with adequate details and transparency. We also provided a good balance between narrative explanations and participants’ quotes or stories to report the findings of this study.

We started coding the participants’ experiences with digital interventions to combat IA in young children. We analyzed their experiences based on three themes: (1) interventions they recognized to combat IA in young children (eg, “Google Family Link”), (2) interventions used by parents or recommended by therapists to their clients (eg, “Apple Parental Control”), and (3) features used or recommended by the participants and how they used them (eg, “device use monitoring”).

The participants’ perceptions of digital interventions were coded and grouped into 3 main themes: advantages, limitations, and recommended functions. We coded the advantages they perceived from the interventions they had known (eg, “filtering inappropriate content”). The limitations came from their experiences using or recommending digital interventions (eg, “complicated to use”). In addition, the limitations were expressed as part of the reasons why some participants chose not to use the existing digital interventions (eg, “data privacy issue”). The recommended functions were analyzed based on the functions or features considered useful by the participants (eg, “suggesting substitutive activities”).

### Ethical Considerations

Ethics approval for this study was obtained from the Swinburne University Human Research Ethics Committee (reference: 20237278-16490; approval date: August 24, 2023). Written informed consent was granted by all participants involved in this study. We did not collect the personal identity of the participants, such as their names or addresses. Each participant was assigned a unique identification number to ensure their anonymity. Each participant in this study was given a voucher for 300,000 Indonesian rupiah (Aus $30 or US $20) in recognition of their participation.

## Results

### Overview of the Internet Use by the Children of the Participating Parents

The parents who participated in this study had given their children access to the internet since early childhood (age of 1-5 years). However, children started using the internet routinely when entering preschool or primary school (age of 5-7 years). Most children (18/22, 82%) had their own mobile devices (eg, smartphones or tablets), whereas the others had to borrow them from their parents or use them collectively with their siblings. All children of the participants were active users of mobile devices, such as smartphones or tablets, and some of them also actively used televisions (18/22, 82%) and nonmobile devices such as laptops or PCs (6/22*,* 27%) and PlayStation consoles (2/22, 9%). Their favorite digital activities were watching videos (eg, YouTube, TikTok, and television channels) and gaming (eg, Minecraft, Roblox, Mobile Legends, and Free Fire). All parents agreed that the internet can be beneficial for their children in terms of education and entertainment. Most parents (17/22, 77%) also said that digital devices helped them fill their children’s free time and keep the children calm and quiet at home. In addition, parents said that some primary schools had started teaching internet technology and delivering school materials through web-based media.

In general, parents showed awareness of the fact that the excessive and uncontrolled use of the internet could bring harmful consequences to their children. Parents whose children had mild to moderate IA risks based on PCIAT scores conveyed various negative impacts of excessive use on their children. This included decreased school performance or creativity (10/22, 45%), inappropriate use of language (8/22, 36%), aggressive behavior (8/22, 36%), procrastination of other activities (7/22, 32%), self-isolation (6/22, 27%), anxiety when not using the internet (5/22, 23%), poor communication with the family (4/22, 18%), eating problems (eg, food intake avoidance; 4/22, 18%), and eye problems (eg, decreased vision, swollen eyes, and red eyes; 3/22, 14%).

### Experiences With Digital Interventions

Parents and therapists generally showed diverse experiences with the use of digital interventions (Table 3). Participants in this study mentioned 3 parental control software they had known to combat IA in young children: Google Family Link, YouTube Kids, and Apple parental control. Google Family Link and Apple parental control offer similar parental control features at the operating system level. A total of 68% (15/22) of the parents and 83% (5/6) of the therapists mentioned Google Family Link, whereas Apple parental control was only mentioned by 17% (1/6) of the therapists. In August 2023, approximately 88% of Indonesian internet users used Android devices, and only 11% used iOS devices [62,63]. This might explain why the participants were more familiar with Google Family Link. In addition, this app is widely available on both the Google Play Store and Apple App Store, whereas Apple parental control is only available for devices with the iOS operating system. One parent said the following:

During the pandemic, I gave my child a Samsung tablet because she needed to study online. Since then, I have also tried using that application [Google Family Link]. The app has been available on her tablet since we bought it, so I can use it immediately.

On the basis of parents’ experiences, 18% (4/22) of parents actively used Google Family Link and perceived it as a useful mechanism to manage children’s digital behavior. In total, 50% (2/4) of these parents (whose children had a normal IA risk) used most app features, including monitoring, screen time limitations, and app management. They and their partners were working parents, so they used the features to supervise their children remotely because they could not always be home. They felt that the app was helpful for supporting them in monitoring and limiting their children’s interaction with technology easily. They communicated the rules about the use of the app clearly to their children so that their children could understand the purpose of using those features. One of the parents said the following:

I usually restrict her screen time using Family Link from Google. I’ve been using this app for a long time because it’s been available on my child’s tablet since the beginning. Usually, I use it to set the duration of the application she can use and what time the application can be used. I also set the total duration for her to use the tablet in a day so the tablet will be locked after reaching the limit. I think it’s quite useful because it can help me organize my daughter, mainly because my husband and I are both working, so we can’t monitor her screen time continuously.

In total, 50% (2/4) of these parents (whose children had mild and moderate IA risks) only used the screen time monitoring and download management features. Through the download management feature, they have to provide parental permission if their children want to download a new app. They used screen time monitoring to understand what apps their children accessed and how much time children spent on each app. However, they only used the feature occasionally (eg, once a week or once a month), and they did not make essential decisions based on screen time monitoring. One of them said the following:

I use the one from Google. It’s called Family Link, as I remember. I organized it so my child had to ask me for permission whenever she wanted to download a new application...I never knew and never used the other features.

Another 14% (3/22) of the parents had used Google Family Link in the past but no longer used it because they found it difficult to make appropriate rules for their children and set them up consistently.

YouTube Kids is a child-friendly version of YouTube that features content for children and has some parental control features (Table 3). However, those features can only be used within the YouTube environment. A total of 45% (10/22) of the parents mentioned and used this app, and 33% (2/6) of the therapists mentioned this app as a way to filter inappropriate videos for children. Among the existing digital interventions, YouTube Kids was one of the most popular because all participants’ children spent most of their screen time watching videos on YouTube or playing games. They perceived that filtering inappropriate videos on YouTube was one of the most important things to prevent deviant behavior in their children. One parent said the following:

She consumes some inappropriate content in YouTube Shorts, even though she doesn’t need that information or content. Some explicit pornographic content also appears in videos, even if it’s intended for children. I really appreciate features on YouTube to filter such videos. This is really important because I can’t always see what my daughter watches.

Some parents actively used parental control features on YouTube to filter inappropriate videos for children (10/22, 45%) and limit access to YouTube (3/22, 14%). However, 18% (4/22) of the parents said that sometimes their children did not like the available child-friendly videos on YouTube Kids, so they tended to find content on other apps such as Google or social media (eg, TikTok and Instagram). One of them said the following:

My child now doesn’t want to use YouTube Kids because he can’t find interesting videos there. Maybe he doesn’t like videos for children anymore. Now, he is more interested in short videos from YouTube shorts or TikTok. He often watches short videos on TikTok using my account because I don’t allow him to create his own account.

In total, 50% (3/6) of the therapists actively recommended the use of parental control software to help parents control their children’s internet use. One of them said the following:

I think it can help parents. In my opinion, parents need help to make their job easier in supervising their children. Such software can be utilized if parents are willing and capable of learning how to use it.

However, they did not recommend it to all clients because they thought that some parents may not have the adequate willingness, capability, or life circumstances to use it. While they knew about Google Family Link and YouTube Kids, they did not recommend which app to use specifically. The other 50% (3/6) of the therapists reported that they sometimes mentioned parental control software as an option for parents to control their children’s behavior but they never recommended it. One of them said the following:

I tell them there are such apps [parental control software]. However, I don’t really understand what applications can be used because I don’t understand technology well. I just advise parents to try such applications, but personally, I never try it myself.

Parents perceived that the digital interventions mentioned in Table 3 could be beneficial in supporting the supervision of their children remotely (11/22, 50%) and filtering inappropriate content for their children (15/22, 68%). Similarly, 67% (4/6) of the therapists also expressed those benefits. One of them said the following:

This [digital interventions] can make it easier for families to monitor how long their children play [digital devices] or what kind of applications are safe to be used. It’s very useful, but many people don’t know about this. We need to let them know so they can monitor their children remotely.

In total, 50% (3/6) of the therapists also perceived the benefit of digital interventions in providing continuous supervision. One of them said the following:

Sometimes parents are limited and busy with their own business, so they cannot control their children for 24 hours a day. Applications like this [parental control apps] will definitely help parents control their child continuously.

In addition, the therapists believed that digital interventions could provide creative ways to educate parents and children on healthy internet use and support the work of IA practitioners such as themselves. With a limited number of mental health workers and low awareness of healthy internet use in Indonesia, this type of intervention may help their work in raising awareness and promoting healthy digital behavior in Indonesian children. One therapist said the following:

In the app, we may provide education about parental digital literacy that can help our work. Interesting education via digital devices will be more popular and exciting than conventional education like seminars or classes that we usually do.

**Table 3:** Summary of the digital interventions known and used by parents and therapists.

Attribute	Parents	Therapists
Interventions known	Google Family Link^a^ Key features: Screen time monitoring Screen time limitations App management App restrictions Content restrictions Location tracking YouTube Kids^b^ Key features: YouTube content filtering YouTube access limitation	Google Family Link^c^ Key features: Screen time monitoring Screen time limitations App management App restrictions Content restrictions Location tracking YouTube Kids^d^ Key features: YouTube content blocking YouTube access limitation Apple parental control^e^ Key features: Screen time monitoring Screen time limitations Download management App restrictions Content restrictions
Interventions used by the parents or recommended by the therapists	Google Family Link^f^ Features used: Screen time monitoring^g^ Screen time limitations^h^ App management^f^ App restrictions^h^ Content restrictions^h^ YouTube Kids^b^ Features used: YouTube content filtering^b^ YouTube access limitation^g^	Parental control apps in general^i^ Features recommended to their clients: Screen time monitoring^i^ Screen time limitations^d^ App restrictions^d^ Content restrictions^i^
Perceived advantages of digital interventions	Supporting parents in monitoring and supervising their children remotely^j^ Filtering inappropriate content^a^	Supporting parents in monitoring and supervising their children remotely^k^ Filtering inappropriate content^k^ Providing continuous supervision^i^ Providing creative ways to educate parents and children^d^ Overcoming the lack of internet addiction practitioners in Indonesia^d^

^a^68% (15/22) of parents.

^b^45% (10/22) of parents.

^c^83% (5/6) of therapists.

^d^33% (2/6) of therapists.

^e^17% (1/6) of therapists.

^f^18% (4/22) of parents.

^g^14% (3/22) of parents.

^h^9% (2/22) of parents.

^i^50% (3/6) of therapists.

^j^50% (11/22) of parents.

^k^67% (4/6) of therapists.

### Limitations of the Existing Digital Interventions

Although most participants recognized the existence of digital interventions to encourage healthy digital behavior in their children, the existing interventions were not highly used. Both parents and therapists perceived that digital interventions might be beneficial to encourage healthy internet use and combat IA in young children. However, they also highlighted some limitations with the existing interventions, which may discourage some of them from using the interventions (Textbox 1).

**Limitations of the existing interventions.**

**Parents**

- Parents finding it difficult to use and set up the app (12/22, 55%)
- Parents being unable to set appropriate rules (9/22, 41%)
- Incompatibility with family culture (4/22, 18%)
- Jeopardized parent-child relationships (4/22, 18%)
- Data privacy issues (2/22, 9%)

**Therapists**

- Parents finding it difficult to use and set up the app (4/6, 67%)
- Parents being unable to set appropriate rules (3/6, 50%)
- Incompatibility with family culture (2/6, 33%)
- Functions not comprehensive (3/6, 50%)
- Children’s privacy issues (2/6, 33%)

The most frequent limitations expressed by the parents and therapists were associated with the parents’ capability and knowledge of how to use the software and create appropriate rules through the interventions. A total of 55% (12/22) of the parents expressed their limitations in using the software. One said the following:

The problem is that applications like that [parental control software] seem complicated. Honestly, I’m a mother who doesn’t really understand technology.

Similarly, 67% (4/6) of the therapists perceived that some Indonesian parents might not be capable of using and willing to use parental control software. In total, 33% (2/6) of the therapists also had difficulties in learning how to use the digital interventions. One said the following:

I tell them [clients] that there is parental control software as an option. However, because I don’t really understand the technology well, I advise parents to try such applications, but I can’t teach them how to use it.

A total of 41% (9/22) of the parents expressed their limitations in creating appropriate rules through the interventions. One said the following:

Even if I can control my child through the app, I have to learn what kind of restrictions should be applied to him

her child

Similarly, 50% (3/6) of the therapists were concerned that parents with low digital literacy and capability would find it difficult to use the features. Although the features are helpful, parents might still be confused about setting appropriate rules for their children. One of them said the following:

We might be able to limit screen time, but the application can only function well if parents set it properly. The question is whether the parents can use it correctly or not.

Most of their clients were also confused about finding proper substitutive activities after limiting their children’s screen time.

A total of 18% (4/22) of the parents and 33% (2/6) of the therapists were also concerned about the apps’ incompatibility with family culture in Indonesia. They reported that some content and tips suggested by the existing interventions did not suit their social values and beliefs. For example, one parent said the following:

Once, I used the feature to filter child-friendly videos because my child really likes watching videos on his tablet. However, I found videos that, sorry to say this, promoting LGBT, which is completely unacceptable because it is not appropriate with our religion and culture.

Parents shared other concerns about using digital interventions. A total of 18% (4/22) of the parents thought that the features of the existing interventions might make children uncomfortable, thus jeopardizing the relationship between parents and children. One parent said the following:

In the past, I wanted to use an application called [Google] Family Link, but my husband and I decided not to use it for some reasons. We want to give more trust to my daughter because she might feel pressured if she feels like she is always being watched.

In total, 9% (2/22) of the parents said that they were worried about using parental control software because they had to synchronize multiple devices and input personal data. As some interventions have app management features, they also worried that the system could access and remove confidential data on their devices. One parent said the following:

I have to connect my device to my child’s device, and that application can delete or move applications on my child’s device. If they can do that, I’m afraid that they may also access or even delete my personal data.

Therapists reported other limitations of the existing interventions in terms of limited functions and trust issues in children. They highlighted that some essential functions are needed in digital interventions. One said the following:

Many things must be improved to be truly helpful, and they [digital interventions] must be made functionally more holistic. For example, they can provide education for parents and children, personalized supervision for parents, and child-friendly content recommendations that are interesting.

Related to the trust issues, one therapist said the following:

Parents might be over-worried and end up using the app excessively. For example, they spy and track a child’s phone without proper communication. No matter what, children aged seven or above need to be given some privacy.

Therapists also had concerns that some children might trick the system if their parents could not provide proper understanding and communication about the rationale behind their supervision through digital interventions such as parental control software. This would make the parents falsely feel that everything is under control.

### Recommended Functions of Digital Interventions

The participants recommended some functions that may be useful in combating IA in young children (Textbox 2). Parents and therapists suggested some functions for a digital intervention, such as supporting parental supervision, suggesting substitutive activities, monitoring and limiting internet use, and suggesting child-friendly content. One parent said the following:

Many parents don’t direct their children to do other activities, so the children will get bored if they don’t spend time with their gadgets [digital devices]. Parents must be able to direct their children to other activities that are positive and interesting for children. It would be helpful if the app could help the parents with that.

**Recommended functions.**

**Parents**

- Supporting parental supervision (14/22, 64%)
- Suggesting substitutive activities (9/22, 41%)
- Monitoring and limiting use (9/22, 41%)
- Suggesting child-friendly content (8/22, 36%)
- Supporting parental decision-making in regulating children’s digital behavior (4/22, 18%)

**Therapists**

- Supporting parental supervision (3/6, 50%)
- Suggesting substitutive activities (3/6, 50%)
- Monitoring and limiting use (3/6, 50%)
- Suggesting child-friendly content (3/6, 50%)
- Developing children’s competencies to combat addictive behavior (5/6, 83%)
- Improving parental digital literacy (4/6, 67%)
- Supporting parents in communicating internet use rules (2/6, 33%)

One therapist also discussed the need for some functions:

In my opinion, parents need help to make their job easier in supervising their children. An application might help. For example, it can recommend good content for children, monitor the device use in real-time, and make children stop playing smoothly.

Parents expected that the interventions would simplify their efforts in making essential decisions regarding education and rules for their children. This is because many parents were confused about regulating their children’s internet use although they knew their children used the internet excessively. They were also afraid that their rules would make their children uncomfortable or offended. One said the following:

I’m often confused with the daily decision I should make about regulating my child. It’s really difficult to handle. I want to give him the internet to make him happy, but I need a clue on how best to control him all the time.

Therapists perceived that parental control software might help supervise children’s digital activities to support behavior change in children. However, they said that parents and children cannot solely rely on screen time limitations or device restrictions to address the problem. One therapist said the following:

What is more important is how parents understand the rules, communicate the rules, and make children understand the rules. Therefore, I advise parents that we cannot completely depend on limitations and blocking through the apps.

In total, 67% (4/6) of the therapists also suggested the need for digital interventions to have comprehensive coverage in managing their children’s internet use, such as across devices or apps. One said the following:

I think it would be more useful if we could limit them at the device [operating system] level. Restrictions on single application become less effective because children can use more than one application.

Therapists highlighted the need for other essential elements to combat IA in children: proactive parental supervision, developing children’s competencies to combat addictive behavior, effective parent-child communication, proper education on healthy internet use, and enjoyable real-world activities. One therapist said the following:

I think a comprehensive intervention is needed. For example, there may be education, real-time measurement, and the ability to carry out addiction prevention over time.

Another therapist had an interesting argument on developing children’s competencies:

The family factors can prevent young children from addiction, like developing children’s foundations [to combat addictive behavior], good communication, and happy life. We should realize that one similar software may cause a different response. Some children may be addicted to it, but not the others. Therefore, preventing addiction will be more powerful if the parents build their children’s foundations from the beginning. With good foundations, children will not easily become addicted when given negative stimulus from the internet.

## Discussion

### Experiences With Digital Interventions to Combat IA in Young Children

In this study, we explored the experiences, perceptions, and considerations of parents and therapists regarding digital interventions to combat IA in young Indonesian children. This study investigated multiperspective views from parents, who are the primary guardians of young Indonesian children, and child therapists, who have expertise and experience in working with children with IA risks. The participating parents and therapists generally perceived that digital interventions such as parental control software could increase parents’ capability to promote healthy digital behavior in their children in the long term. This perception aligned with the DBCI concept that using behavior change principles in digital interventions could be useful to promote healthy behavior in their users [40]. In this case, promoting healthy digital behavior was considered beneficial to combat IA in young children. Other studies have also highlighted the similar potential of DBCI use for addressing the problem, and the current interventions primarily focus on screen time regulation [24,25].

The participants similarly perceived the advantages of using digital interventions for combating the problem, such as supporting parents in monitoring and supervising their children remotely (15/28, 54%) and filtering inappropriate content for children (19/28, 68%). In addition, the therapists perceived more benefits, such as facilitating continuous parental supervision (3/6, 50%) and supporting health practitioners’ work to promote and educate parents and children on healthy internet use (2/6, 33%).

Almost all participants (26/28, 93%) highlighted the role of parents as the key to developing healthy or risky digital behavior in their children, especially in children aged <12 years. This is because parental influence and mediation significantly impact children’s digital behavior [22,42,56]. In the context of young children, no one is more influential than parents, although other parties can also influence them (eg, siblings and peers). Parents provide their children with the internet, so they are responsible for managing their children’s internet use [55]. In addition, children aged <12 years are generally still in the cognitive development phase [64], so they may not have good self-regulation and self-efficacy [65]. However, parents often, intentionally or not, expose their children to risky digital behavior [55,66]. The findings showed that many parents (13/22, 59%) were confused about educating their children and regulating their healthy digital behavior. As digital parenting is something new, they may not necessarily be able to regulate their children’s internet use well even though various monitoring and restricting features are available [56]. Therefore, the interventions may not only intervene with children’s use of technologies but also with parents’ management of their children’s technology use. The interventions should be able to support and educate parents on how to encourage their children’s healthy digital behavior effectively.

Participants in this study recognized 3 digital interventions to combat IA in the form of parental control software: Google Family Link, Apple parental control, and YouTube Kids. Despite the positive sentiment on the potential of digital interventions, the use of the existing features of those systems among the participants was not convincing. This is because, of the 28 participants, only 4 (14%) parents actively used Google Family Link, 10 (36%) parents used YouTube Kids, and 3 (11%) therapists actively recommended using parental control software to their clients. In addition, the parental control features on YouTube Kids can only monitor and limit children’s internet activities while watching YouTube content. Therapists argued that the regulations at the app level would be less effective because children typically interact with more than one app, device, or streaming service. This statement was supported by the findings that all children of the participating parents used more than one app daily (eg, YouTube, Minecraft, and TikTok) and many parents (18/22, 82%) gave their children access to more than one device (eg, smartphone, tablet, and television).

A total of 50% (2/4) of Google Family Link users used all features except location tracking. Their children had normal addiction risks based on the PCIAT score, and they reported the usefulness of the app in helping them supervise their children when the parents were busy with their activities. This may be initial evidence that the proper use of digital intervention features could be helpful for parents in combating IA in their children. However, we need further evidence on the mechanism of the app in preventing or reducing IA in children. Another 50% (2/4) of Google Family Link users only used 2 features of the app: screen time monitoring and parental permission to download new apps. However, they reported that they only monitored children’s screen time occasionally (eg, once a week or once a month) and did not make essential decisions based on monitoring. Therefore, we could not explore their overall experiences with using the app.

### Limitations of Digital Interventions to Combat IA in Young Children

The participants expressed some limitations that may underlie the lack of use of the existing digital interventions. The limitations might also decrease participants’ motivation to use digital interventions. Both parents and therapists mentioned 3 similar limitations with these interventions: the parents’ difficulties in using the apps, the parents’ difficulties in setting appropriate rules through the apps, and the incompatibility of the features or content with family culture in Indonesia.

Parents and therapists highlighted that one of the most significant obstacles in combating IA in children are the parental limitations in terms of capability and time to educate their children and manage their digital behavior. This parental issue could be the main barrier to using digital interventions for supporting internet use parenting [67,68]. Therefore, the interventions should support and simplify parental efforts to manage their children’s digital behavior, as suggested in the UTAUT model [46]. The capability of computer systems in digital interventions should be used to support, assist, and simplify parental efforts in sustainably educating their children and regulating their digital behavior. If the intervention requires many complex settings and actions, parents may feel that it will add load and complexity to their lives. No matter how good the features are, the intervention will be less practical if the potential users have no intention of using it and capacity to use it. We cannot assume that all parents have adequate digital literacy and capability [69]. Thus, it is essential to match the interventions to parents’ digital capabilities, capacity to engage, and other limitations. In addition, the therapists were also worried that children would outsmart or work around the regulations or restrictions, leading parents to mistakenly feel that everything is under control. Therefore, the interventions should be designed to include a range of scenarios and conditions so that children cannot work around or override them [70].

A total of 21% (6/28) of the participants in this study perceived that the existing digital interventions might not suit some families’ cultures in Indonesia. For example, they reported that some child-friendly content suggested on YouTube Kids was inappropriate based on their own cultures and beliefs. In other cases, parents felt that the screen time restriction feature might elicit the impression of distrust toward their children, so they felt uncomfortable using it. Considering the culture of the users is very important when developing digital health interventions for children [71-73]. Reflecting on such cases, it is crucial to consider the potential users’ cultures in developing digital interventions to combat IA.

Parents expressed concern that using limitation or restriction features may lead to negative experiences for children and jeopardize parent-child relationships. Other studies have also highlighted that healthy parent-child relationships are essential in combating IA [74-76]. Therefore, it is important to consider that the interventions should not harm the relationship between parents and children. For example, the system may assist parents in communicating the rules appropriately so that their children can understand and accept them well. Providing a positive experience to the parents is also essential to avoid any frustration so that they can provide better supervision and communication to their children. For example, we could facilitate parents’ experience of the benefits of their efforts through the interventions or create engaging gamification features that simplify evidence-based information and support parents in establishing rules for their children in novel and attractive ways (eg, short videos, animations, and role-playing).

Concerns about data privacy and security were also reported by 9% (2/22) of the parents due to the nature of parental control software systems. The software typically has privacy warnings and agreements presented to its users [77,78], but parents may still not understand or be confident in using it. In addition, related studies have reported privacy problems with the current parental control software, such as accessing personal data and sharing user data with third parties without appropriate consent and transparency [79,80]. Therefore, further studies are needed to investigate how the developers overcome this privacy concern and how those privacy issue warnings and agreements are delivered to ensure that the users feel confident and secure in using the software.

Therapists perceived other limitations in terms of functional and children’s privacy issues. In total, 50% (3/6) of the therapists highlighted some essential functions not covered in the existing interventions, such as proactive parental supervision, children’s competencies to combat addictive behavior, parent-child communication enhancement, proper education on healthy internet use, and enjoyable real-world activity suggestions. Other studies have also reported the absence of similar functions in the existing parental control software, such as maintaining family relationships, parental mediation, and social support [70,81].

A total of 33% (2/6) of the therapists also emphasized that the need for the use of digital interventions should not raise any privacy issues in children, which can jeopardize the parent-child relationship. Parents should be responsible for protecting children’s privacy in web-based environments [82]. However, the therapists had experiences with parents becoming overprotective since using parental control software. They reported rare cases in which their clients (parents) seemed to be overmonitoring their children almost all the time, which could create other family relationship problems. This issue has not been discussed much in the context of digital health interventions for children. Therefore, we need further research studies to investigate how this issue affects children and how to address the issue appropriately.

### Recommended Functions for Digital Interventions to Combat IA in Young Children

The participants in this study recommended several functions for digital interventions to combat IA in young children. They suggested some functions that were covered in the interventions discussed in this study, such as parental supervision (17/28, 61%), use monitoring and restrictions (12/28, 43%), and child-friendly content filtering (11/28, 39%). This means that those functions should be maintained and improved in the interventions. However, the participating parents expressed some suggestions to improve the implementation of those functions.

One of the main parental limitations identified in this study was the parents’ confusion or inability to determine and enforce appropriate supervision for their children. In line with this problem, 18% (4/22) of the parents recommended decision-making support features to guide them in monitoring and creating appropriate rules for their children. This feature may complement and improve the monitoring and restriction functions that already exist in the interventions. We may adopt the concept of decision support system software in the context of promoting healthy digital behavior in young children [83]. The digital interventions may also capture and use some valuable data from the children (eg, screen time, web-based activities, and favorite content) as input to provide tailored or personalized decision-making suggestions for the parents [84].

Regarding use restrictions, both parents and therapists similarly expected the system not to jeopardize the relationship between parents and children. Providing digital interventions with proper education and suggestions may help parents communicate the rules better so that their children can understand and accept them properly. For the content filtering function, the participants expected that the suggested content would be appropriate for their culture, social values, and beliefs. Providing culture-specific or personalized features for the users may help address their expectations [85,86].

The participants also recommended functions that might not exist in the interventions discussed. In total, 41% (9/22) of the parents and 50% (3/6) of the therapists recommended the need to suggest substitutive activities for children. Parents often feel confused about providing proper and positive activities for their children apart from internet activities. Some parents also relied on the use of digital devices as an option to fill their children’s free time. Therefore, to prevent internet overuse, it would be helpful if the system could suggest alternative activities that suit the needs and preferences of their children. The activities suggested should be attractive enough for children to shift from the virtual to the physical world [87].

Therapists highlighted that digital interventions should be used to develop children’s competencies in understanding internet use properly to combat addictive behavior. According to the IT-CPU theory, children’s behavior and attitude toward internet use can significantly influence their IA risks [7]. Therefore, developing children’s competencies to combat addictive behavior may help prevent or reduce IA risks in young children. In total, 67% (4/6) of the therapists also emphasized the need to improve digital literacy levels in children and parents. This is supported by previous studies that reported the significant contribution of improving digital literacy through education to the success in combating IA in children and adolescents [69,88,89]. Digital interventions were seen as potential tools to provide education in more interesting ways (eg, gamified learning) and increased accessibility [90,91].

In this study, parents were concerned that regulating internet use through the system would jeopardize their relationship with their children. This is also supported by the IT-CPU theory that the parent-child relationship is one of the maintaining factors that influence the IA risk in children [7]. In line with this concern, 50% (3/6) of the therapists expressed the need for digital intervention features to support parents in communicating internet use rules to their children. It is important to consider how the interventions may help parents not only set the rules but also deliver and communicate them to the children properly. The use of gamification features (eg, turning the rules into missions with accomplishment rewards and using animated videos or avatars to explain the importance of the rules) may help make it easier for children to understand and accept the rules [42,92,93].

### Implications for Future Digital Interventions

Reflecting on the findings of this study, some implications can be derived related to the design of digital interventions and other aspects that influence the use and acceptability of digital interventions to combat IA in young children. The participants in this study expressed some limitations of the key features of the existing interventions (eg, screen time limitation, screen time monitoring, and content filtering) that discouraged them from using the interventions. Nevertheless, they still perceived those features as helpful for combating IA in children. For example, parents may limit children’s screen time through the system but still have difficulty providing substitutive activities. In other cases, parents can monitor children’s screen time, but they may still be confused about creating and communicating the appropriate rules based on monitoring. Therefore, this study suggests the need to improve the existing features to be more usable and relatable for the parents to increase their motivation and capability to use the interventions.

According to the UTAUT model, the acceptability of the interventions will be better if we can provide functions that are perceived as beneficial by the potential users [46]. The findings of this and other related studies highlight that the existing features in digital interventions (eg, screen time monitoring and limitations) might not be sufficient to combat IA in young children [33,70]. Our findings suggest that digital interventions should focus not only on restricting and monitoring screen time but also on suggesting substitutive activities for children, developing children’s competencies to combat addictive behavior (eg, attitude toward internet use and self-regulation), improving digital literacy in children and parents, and supporting parental decision-making to promote healthy digital behavior in their children. To provide continuous and comprehensive intervention, it is also essential to develop interventions that can cover all the devices or apps that children use. For example, we may develop the interventions to have control over the entire device (operating system) or across devices as children may use more than one app or device.

The appropriateness of the features or content provided by digital interventions could also determine the users’ perception, engagement, and acceptability [46,94]. The findings of this and other digital health intervention studies report similar concerns that some features or content provided by the interventions might be inappropriate for the users’ culture or beliefs [72,73]. Participants in this study recognized and used the existing interventions developed by big technology multinational companies (eg, Google, YouTube, and Apple). Therefore, the features or content provided by the digital interventions may need to be adjusted to suit their unique characteristics and culture.

To our knowledge, no digital intervention has been developed for the Indonesian context to date. Given the higher prevalence of IA in lower-income regions such as Indonesia [35,36], further studies may be needed to develop culture-appropriate digital interventions for vulnerable populations in these regions. In addition, we suggest adopting tailoring or personalization mechanisms to deliver suitable features or content based on the users’ characteristics and culture. These mechanisms can also be beneficial to increase behavior change intention in combating addictive behavior [27,95].

Parents play an important role in supervising and educating children to combat IA [70]. This explains why existing digital interventions for young children typically involve parents in combating IA [52]. In this study, we found that parents perceived barriers to using the existing digital interventions due to their limitations in terms of capability, knowledge, and skills to use the interventions and create appropriate rules. These limitations emphasize the need to provide adequate training for parents to increase their motivation and capability to use digital interventions [96,97]. Collaborating with parents in designing digital interventions may also be beneficial to understand their limitations and suit their needs [98]. In addition, we may support parents through gamification features, such as goal setting, progress tracking, rewards and punishments, and visualization of the results of their supervision, to enhance their ability and engagement in supervising their children [99,100].

Privacy issues related to the use of digital interventions cannot be overlooked. The acceptability of digital interventions may decrease due to parents’ distrust of data security and the findings of other studies that have reported privacy violation cases by parental control software [79,80]. Although the interventions typically have privacy warnings and agreements [77,78], we should ensure that the parents can understand and accept the provisions properly; otherwise, they will be reluctant to use the interventions. In addition, providing education on this issue may help parents feel more confident in using the interventions. Another privacy issue raised was related to children’s privacy problems due to excessive parental supervision (eg, overmonitoring or being overprotective). Although there is a lack of discussion of this issue in the context of internet use, therapists emphasized the need to consider this in future interventions to avoid other family problems. We argue that improving parents’ knowledge of digital parenting and assisting their decision-making through digital interventions may help address this issue.

### Limitations of This Study

This study has several limitations. This study may have limited generalizability as we used a small number of participants and a nonrandom sampling strategy (convenience sampling). Therefore, reader discretion is needed in considering the context of this study when using or applying the findings.

In this study, we involved parents and therapists as the children’s stakeholders in combating IA, but we did not collect data from children. Therefore, further studies may be needed to complement the results of this study by exploring children’s experiences with and perceptions of related topics.

To achieve the objective of this study, we focused on exploring participants’ experiences and perceptions regarding the digital interventions already available in Indonesia. Other digital interventions might have been developed in other contexts or regions, but we did not include them in this study. Further studies may be needed to investigate other interventions not included in this study.

Most participating parents (20/22, 91%) in this study were mothers or female individuals. This is because, in the culture of most Indonesian families, the mother typically has a role as the primary guardian of the children. As we required the participation of the primary guardians of children, we did not prioritize an equal proportion of mother and father participation. Nevertheless, the children of the participating parents had quite a balanced gender proportion (64% boys and 36% girls). In addition to this, we found no significant differences between mothers’ and fathers’ perspectives in this study.

### Conclusions

This study shed light on the experiences, perceptions, and considerations of parents and therapists regarding the use of digital interventions for combating IA in young Indonesian children. Participants in this study perceived the benefits of digital interventions in continuously promoting healthy digital behavior in young children and supporting parents in regulating their children’s internet use. However, the participants did not highly use the existing interventions due to some limitations. This includes essential issues such as the interventions’ functionality and usability, parental capability, cultural incompatibility, parent-child relationships, and privacy.

Our findings suggest that digital interventions should focus not only on restricting and monitoring screen time but also on suggesting substitutive activities for children, developing children’s competencies to combat addictive behavior (eg, attitude toward internet use and self-regulation), improving digital literacy in children and parents, and supporting parental decision-making to promote healthy digital behavior in their children. Suggestions for future digital interventions are provided, such as making the existing features more usable and relatable, investigating gamification features to enhance parental motivation and capability in managing their children, providing tailored or personalized content to suit users’ characteristics, and considering the provision of training and information on the use of interventions and privacy agreements.

This study contributes to extending the knowledge from parents’ and therapists’ perspectives on the current state, existing perceptions, and future implications of digital interventions to combat IA in young Indonesian children. The findings of this study will be valuable considerations in evaluating the existing interventions and developing better interventions in the future. For future work, we aim to collaborate with multiple stakeholders (eg, parents, children, teachers, peers, and therapists) to develop digital interventions to combat IA in young children by continuously encouraging healthy digital behavior and improving parental mediation of children’s internet use. The findings of this study will be the primary considerations for future work in developing digital interventions to prevent or reduce IA risk in children.

## Appendix

Multimedia Appendix 1 COREQ (COnsolidated criteria for REporting Qualitative research) Checklist.

Multimedia Appendix 2 Semistructured Interview Questions.

## Abbreviations

COREQ: Consolidated Criteria for Reporting Qualitative Research
DBCI: digital behavior change interventions
IA: internet addiction
IT-CPU: Interactional Theory of Childhood Problematic Media Use
PCIAT: Parent-Child Internet Addiction Test
RQ: research question
UTAUT: Unified Theory of Acceptance and Use of Technology
