# Supplementary material for: Digital Interventions for Combating Internet Addiction in Young Children: Qualitative Study of Parent and Therapist Perspectives
Source: JMIR Pediatr Parent. 2024 Apr 26;7:e55364. doi: 10.2196/55364 (PMC11087864; doi:10.2196/55364)
Supplement: Multimedia Appendix 1 [file pediatrics_v7i1e55364_app1.docx]

Appendix 1. COREQ (COnsolidated criteria for REporting Qualitative research) Checklist

| **Topic** | **Item No.** | **Guide Questions/Description** | **Reported on**  **Page No.** |
| --- | --- | --- | --- |
| **Domain 1: Research team and reﬂexivity** | | | |
| *Personal characteristics* | | | |
| Interviewer/facilitator | 1 | Which author/s conducted the interview or focus group? | Page 7–8 |
| Credentials | 2 | What were the researcher’s credentials? E.g. PhD, MD | Page 4–5 |
| Occupation | 3 | What was their occupation at the time of the study? | Page 4–5 |
| Gender | 4 | Was the researcher male or female? | Page 4–5 |
| Experience and training | 5 | What experience or training did the researcher have? | Page 4–5 |
| *Relationship with participants* | | | |
| Relationship established | 6 | Was a relationship established prior to study commencement? | Page 7–8 |
| Participant knowledge of  the interviewer | 7 | What did the participants know about the researcher? e.g. personal  goals, reasons for doing the research | Page 7–8 |
| Interviewer characteristics | 8 | What characteristics were reported about the interviewer/facilitator?  e.g. Bias, assumptions, reasons and interests in the research topic | Page 7–8 |
| **Domain 2: Study design** | | | |
| *Theoretical framework* | | | |
| Methodological orientation and Theory | 9 | What methodological orientation was stated to underpin the study?  e.g. grounded theory, discourse analysis, ethnography, phenomenology,  content analysis | Page 4 |
| *Participant selection* | | | |
| Sampling | 10 | How were participants selected?  e.g. purposive, convenience, consecutive, snowball | Page 5 |
| Method of approach | 11 | How were participants approached? e.g. face-to-face, telephone, mail,  email | Page 7–8 |
| Sample size | 12 | How many participants were in the study? | Page 5 |
| Non-participation | 13 | How many people refused to participate or dropped out? Reasons? | Page 5 |
| *Setting* | | | |
| Setting of data collection | 14 | Where was the data collected? e.g. home, clinic, workplace | Page 7–8 |
| Presence of non- participants | 15 | Was anyone else present besides the participants and researchers? | Page 7–8 |
| Description of sample | 16 | What are the important characteristics of the sample?  e.g. demographic data, date | Page 5–7 |
| *Data collection* | | | |
| Interview guide | 17 | Were questions, prompts, or guides provided by the authors? Was it pilot tested? | Page 7–8, Appendix 1 |
| Repeat interviews | 18 | Were repeat interviews carried out? If yes, how many? | N/A |
| Audio/visual recording | 19 | Did the research use audio or visual recording to collect the data? | Page 7–8 |
| Field notes | 20 | Were ﬁeld notes made during and/or after the interviews or focus group? | Page 7–8 |
| Duration | 21 | What was the duration of the interviews or focus group? | Page 7–8 |
| Data saturation | 22 | Was data saturation discussed? | N/A |
| Transcripts returned | 23 | Were transcripts returned to participants for comment and/or correction? | Page 7–8 |

| **Topic** | **Item No.** | **Guide Questions/Description** | **Reported on**  **Page No.** |
| --- | --- | --- | --- |
| **Domain 3: analysis and ﬁndings** | | | |
| *Data analysis* | | | |
| Number of data coders | 24 | How many data coders coded the data? | Page 8 |
| Description of the coding  tree | 25 | Did authors provide a description of the coding tree? | Page 8 |
| Derivation of themes | 26 | Were themes identiﬁed in advance or derived from the data? | Page 8 |
| Software | 27 | What software, if applicable, was used to manage the data? | Page 8 |
| Participant checking | 28 | Did participants provide feedback on the ﬁndings? | N/A |
| *Reporting* | | | |
| Quotations presented | 29 | Were participant quotations presented to illustrate the themes/ ﬁndings? Was each quotation identiﬁed? e.g. participant number | Page 9–16 |
| Data and ﬁndings consistent | 30 | Was there consistency between the data presented and the ﬁndings? | Page 9–16 |
| Clarity of major themes | 31 | Were major themes clearly presented in the ﬁndings? | Page 9–16 |
| Clarity of minor themes | 32 | Is there a description of diverse cases or discussion of minor themes? | Page 9–16 |

Developed from: Tong A, Sainsbury P, Craig J. Consolidated criteria for reporting qualitative research (COREQ): a 32-item checklist for interviews and focus groups. *International Journal for Quality in Health Care*. 2007. Volume 19, Number 6: pp. 349 – 357.
