# Supplementary material for: Digital Interventions for Combating Internet Addiction in Young Children: Qualitative Study of Parent and Therapist Perspectives
Source: JMIR Pediatr Parent. 2024 Apr 26;7:e55364. doi: 10.2196/55364 (PMC11087864; doi:10.2196/55364)
Supplement: Multimedia Appendix 2 [file pediatrics_v7i1e55364_app2.docx]

Appendix 2. Semistructured Interview Questions

| **Sub-Topic** | **No.** | **Question** |
| --- | --- | --- |
|  | 0 | Can you briefly share about your children's internet activities?  [for parents only] |
| Experience | 1 | Can you share your experience in managing the internet activities of your children/clients? |
|  | 2 | Can you share your experience preventing or reducing Internet Addiction risks in your children/clients? |
|  | 3 | Can you share your knowledge and experience in using digital interventions or parental control software to combat internet overuse in children? |
| Perception | 4 | What do you think about using digital interventions to prevent or reduce Internet Addiction risks in children? |
|  | 5 | What are the benefits of using digital interventions to prevent or reduce Internet Addiction risks in children? |
|  | 6 | What are the barriers or limitations perceived in using digital interventions? |
| Recommendation | 7 | What functions do you find useful from the current digital interventions? |
|  | 8 | What functions would be beneficial from digital interventions to prevent or reduce Internet Addiction risks in children? |
